# SLM2 Is A Novel Cardiac Splicing Factor Involved in Heart Failure due to Dilated Cardiomyopathy

**DOI:** 10.1016/j.gpb.2021.01.006

**Published:** 2021-07-15

**Authors:** Jes-Niels Boeckel, Maximilian Möbius-Winkler, Marion Müller, Sabine Rebs, Nicole Eger, Laura Schoppe, Rewati Tappu, Karoline E. Kokot, Jasmin M. Kneuer, Susanne Gaul, Diana M. Bordalo, Alan Lai, Jan Haas, Mahsa Ghanbari, Philipp Drewe-Boss, Martin Liss, Hugo A. Katus, Uwe Ohler, Michael Gotthardt, Ulrich Laufs, Katrin Streckfuss-Bömeke, Benjamin Meder

**Affiliations:** 1 Department of Cardiology, Angiology and Pneumology, University Hospital Heidelberg, D-69120 Heidelberg, Germany; 2 Klinik und Poliklinik für Kardiologie, Universitätskrankenhaus Leipzig, D-04103 Leipzig, Germany; 3 German Center for Cardiovascular Research (DZHK), Partner site Heidelberg, D-69120 Heidelberg, Germany; 4 Clinic for General and Interventional Cardiology/Angiology, Herz- und Diabeteszentrum NRW, Ruhr-Universität Bochum, D-32545 Bad Oeynhausen, Germany; 5 Department of Cardiology and Pneumology, University Hospital, Georg-August University Goettingen, D-37075 Goettingen, Germany; 6 German Center for Cardiovascular Research (DZHK), Partner site Goettingen, D-37075 Goettingen, Germany; 7 Berlin Institute for Medical Systems Biology, Max Delbrück Center for Molecular Medicine in the Helmholtz Association, D-10115 Berlin, Germany; 8 Institute of Biology, Humboldt Universität zu Berlin, D-10099 Berlin, Germany; 9 Neuromuscular and Cardiovascular Cell Biology, Max Delbrück Center for Molecular Medicine in the Helmholtz Association, D-13092 Berlin, Germany; 10 German Center for Cardiovascular Research (DZHK), Partner site Berlin, D-10117 Berlin, Germany; 11 Stanford Genome Technology Center, Department of Genetics, Stanford Medical School, Palo Alto, CA 94304, USA

**Keywords:** Splicing, Titin, Dilated cardiomyopathy, KHDRBS3, SLM2

## Abstract

Alternative mRNA **splicing** is a fundamental process to increase the versatility of the genome. In humans, cardiac mRNA splicing is involved in the pathophysiology of heart failure. Mutations in the splicing factor RNA binding motif protein 20 (RBM20) cause severe forms of cardiomyopathy. To identify novel cardiomyopathy-associated splicing factors, RNA-seq and tissue-enrichment analyses were performed, which identified up-regulated expression of Sam68-Like mammalian protein 2 (**SLM2**) in the left ventricle of dilated cardiomyopathy (**DCM**) patients. In the human heart, SLM2 binds to important transcripts of sarcomere constituents, such as those encoding myosin light chain 2 (*MYL2*), troponin I3 (*TNNI3*), troponin T2 (*TNNT2*), tropomyosin 1/2 (*TPM1/2*), and **titin** (*TTN*). Mechanistically, SLM2 mediates intron retention, prevents exon exclusion, and thereby mediates alternative splicing of the mRNA regions encoding the variable proline-, glutamate-, valine-, and lysine-rich (PEVK) domain and another part of the I-band region of titin. In summary, SLM2 is a novel cardiac splicing regulator with essential functions for maintaining cardiomyocyte integrity by binding to and processing the mRNAs of essential cardiac constituents such as titin.

## Introduction

Post-transcriptional modification of mRNA is an essential process of transcriptional and translational control and further promotes proteome diversity in eukaryotes. Evaluation of the RNA isoform dynamics reveals that nearly all multi-exonic genes are alternatively spliced in mammals, involving specific regulation of key proteins that form the spliceosome. The basic ribonucleoprotein complex selects exons and removes introns during mRNA maturation [1]. Exon selection and control of splicing activity are achieved by a class of non-spliceosomal RNA-binding proteins (RBPs), which are divided into three subclasses: serine and arginine-rich (SR) proteins, tissue-specific splicing factors, and canonical heterogeneous nuclear ribonucleoproteins (hnRNPs) [2], [3], [4], [5]. Of those, the subclass of tissue-specific splicing factors can either act as activators or inhibitors of alternative splicing, thereby representing dynamic regulatory elements [6], [7], [8].

Tissue-enriched expression in heart and skeletal muscle has been demonstrated for several splicing factors, including RNA binding fox-1 homolog 1 (RBFOX1), RNA binding motif protein 20 (RBM20), and RNA binding motif protein 24 (RBM24) [9], [10]. Given their essential roles for normal cellular function, genetic mutations of alternative splicing factors cause a wide range of diseases including cancer [11], [12], neurodegenerative disorders [13], [14], [15], and cardiovascular diseases [6], [7], [16], [17], [18], [19]. The mechanisms underlying the onset of cardiomyopathy in, *e.g.*, RBM20 deficiency involve incorrect processing of several splicing targets at once, including important contractile and structural proteins, such as calcium/calmodulin-dependent protein kinase II delta (CaMKIId), ryanodine receptor, tropomyosin, troponin, and titin, thereby affecting the structural and functional properties of cardiomyocytes including calcium handling [20], [21], [22].

Our group has previously elucidated the roles of two cardiac splicing factors in cardiomyopathy, including RBFOX1 as a heart failure-related splicing factor and RBM20 as a cause for non-compaction cardiomyopathy [6], [18]. Here, we aimed to search for novel splicing factors involved in the pathogenesis of heart failure. By screening a large cardiac transcriptome dataset of dilated cardiomyopathy (DCM) and non-failing hearts, we identified candidates and evaluated the role of Sam68-like mammalian protein 2 (SLM2) in cardiac mRNA processing. The results suggest SLM2 as a novel splicing factor with implications for human cardiomyopathy and heart failure.

## Results

### *SLM2* expression is increased in the failing left ventricle of DCM patients

In order to identify important RNA splicing factors in the failing heart, we combined data on tissue-enriched expression and disease-associated regulation in human heart muscles. The myocardial expression data from deep transcriptome sequencing of DCM patients (*n* = 33) were compared to that of non-failing controls (*n* = 24) to determine the expressional regulation of mRNA-binding proteins (mRBPs) in the myocardium [23]. The diagnosis of DCM was confirmed after excluding secondary etiologies such as coronary artery disease. This workup included invasive and non-invasive characterization of the participants, to exclude any other heart failure-related disease entity [24].

**Figure 1:**
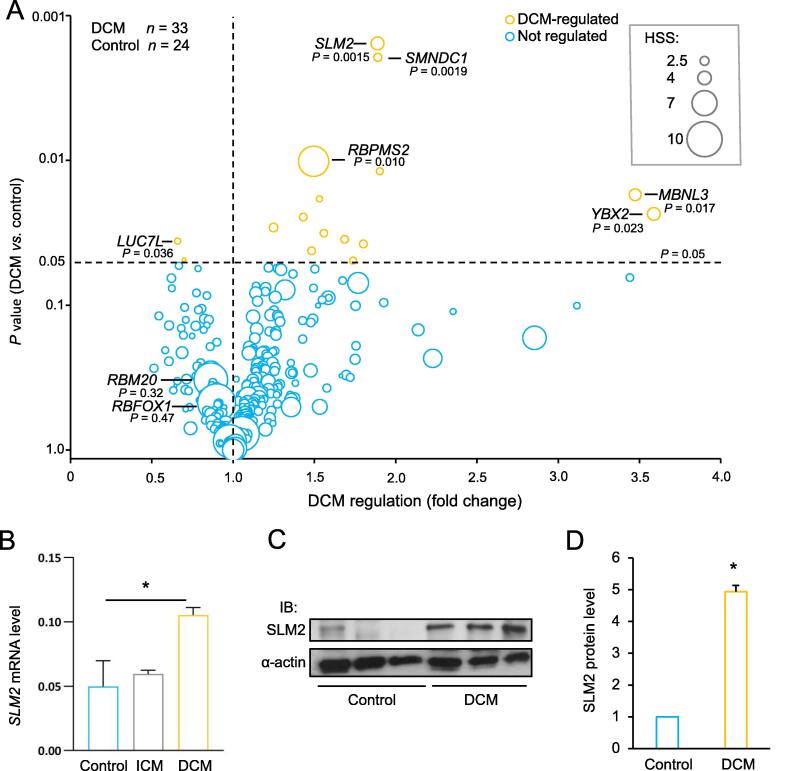
**Regulation and enrichment of mRBPs in failing human hearts** **A.** Regulation of 421 mRPB-coding genes in the heart of patients diagnosed with DCM (*n* = 33) compared to controls (*n* = 24) (Student’s *t*-test). Size of the bubble indicates a HSS (arbitrary unit) in 53 different human tissues from a total of 11,467 human tissue samples. **B.***SLM2* mRNA expression levels in heart tissues of patients (DCM and ICM; each *n* = 3) and controls (*n* = 5). The *SLM2* mRNA expression levels were normalized to the mRNA expression level of *RPLP0*, which served as the reference gene. **C.** Representative IB image depicting the protein expression levels of SLM2 in heart tissues of DCM patients and controls. α-actin served as a loading control. **D.** Densitometric quantification of SLM2 protein expression in heart tissues of DCM patients (*n* = 4) and controls (*n* = 3). α-actin served as a loading control and was used for normalization. *, *P* < 0.05 (Student’s *t*-test). mRPB, mRNA-binding protein; DCM, dilated cardiomyopathy; ICM, ischemic cardiomyopathy; HSS, heart-specificity score; SLM2, Sam68-like mammalian protein 2; IB, immunoblot.

We then combined the data on expressional regulation in the myocardium with heart-specificity scores (HSSs; arbitrary unit) for mRBP-coding genes, which were generated from a total of 11,467 tissue samples from 53 different types of human tissues, including 336 tissue samples from left ventricle [25]. A total of 141 mRBP-coding genes had a HSS ≥ 3. Among the mRBP-coding genes with a high HSS, we found genes coding for well-known heart-disease causing splicing factors, such as *RBM20* (HSS = 10.2) and *RBFOX1* (HSS = 11.4). Despite their high enrichment in myocardial tissue, these two genes were not significantly changed on the expression level in the myocardium of DCM patients. In contrast, we found 14 mRBP-coding genes to be up-regulated, whereas 2 mRBP-coding genes were down-regulated in the DCM patients compared to controls (*P* < 0.05). mRBP-coding genes with broader tissue expression and dysregulation in DCM included the mRNA splicing factor gene *SLM2* (*KHDRBS3* or *T-STAR*) (1.88-fold change, *P* = 0.0015) and RNA binding protein, mRNA processing factor 2 (*RBPMS2*) (1.49-fold change, *P* = 0.010), indicating their potential role in DCM pathogenesis (Figure 1A). We validated the increase of *SLM2* expression in the human heart tissues of DCM patients compared to the controls on mRNA level using qPCR (*P* < 0.05) (Figure 1B) and on protein level using immunoblot (Figure 1C). Quantification revealed a significant increase of SLM2 on protein level in DCM hearts by 5.3-fold (Figure 1D), whereas no change of *SLM2* was seen in the heart tissues of ischemic cardiomyopathy (ICM) patients on mRNA level (Figure 1B). We chose to further characterize the function of SLM2, whose role in cardiomyocytes or heart disease is currently unknown.

### Knockdown of ***slm2*** expression leads to heart failure ***in vivo***

**Figure 2:**
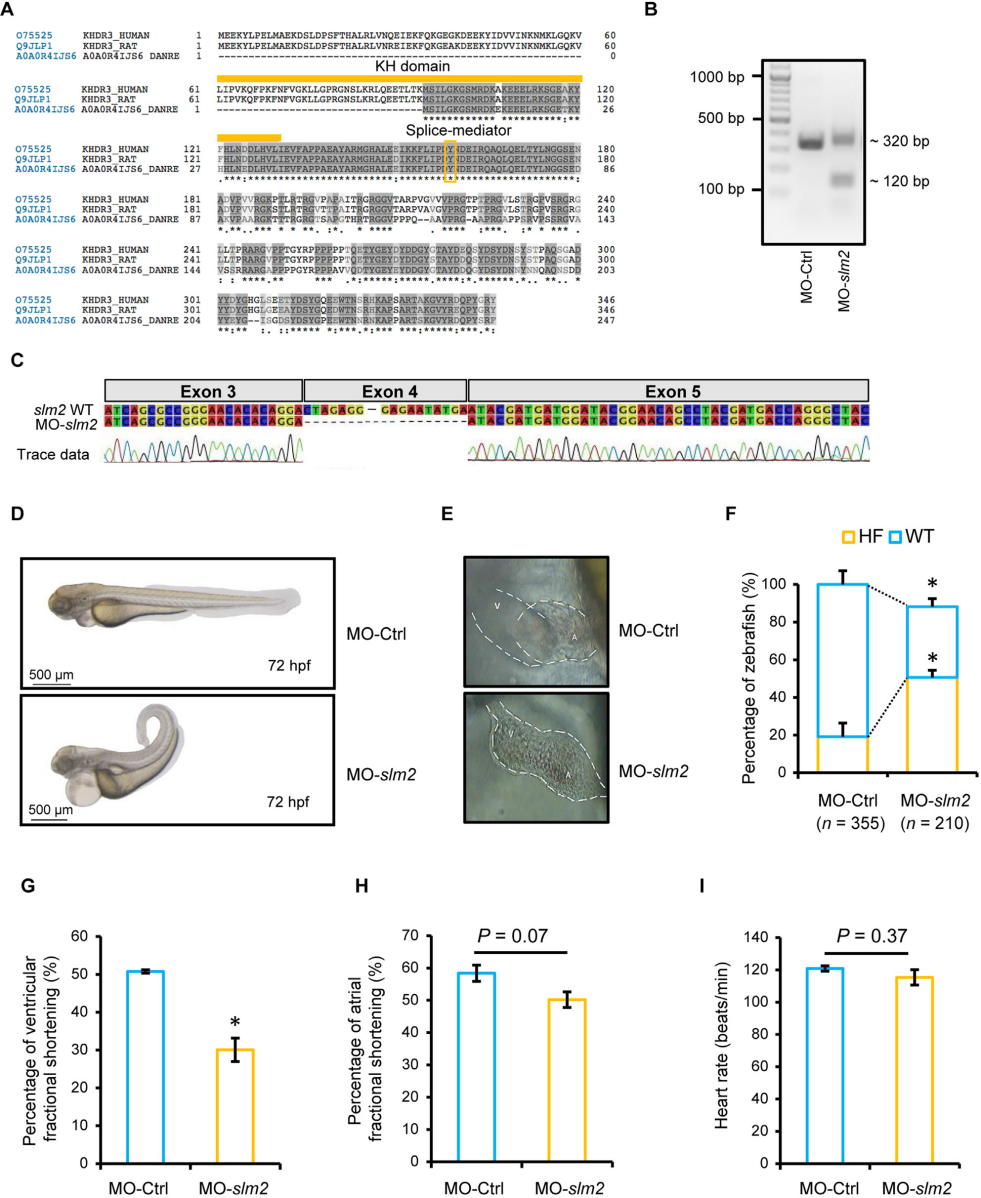
***slm2* knockdown results in heart failure in zebrafish** **A.** Conservation of the SLM2 proteins in humans, rats, and zebrafish. The KH domain which mediates RNA interaction and the tyrosine (Y) residue which mediates splicing are labeled. **B.** PCR analysis of zebrafish injected with splice morpholino targeting *slm2* mRNA (MO-*slm2*) or control MO (MO-Ctrl) at 1–2-cell-stage embryos. The 320-bp product indicates the wild-type *slm2* and the 120-bp product indicates loss of function by exon skipping. **C.** The effectiveness of MO-*slm2* validated by Sanger sequencing of the PCR products shown in (B). **D.** Representative images of zebrafish injected with MO-*slm2* and MO-Ctrl, respectively. Scale bar, 500 µm. **E.** Magnification of the hearts of zebrafish shown in (D). **F.** Quantitative analysis of the phenotypes of zebrafish after MO-*slm2* or MO-Ctrl injection. **G.** Ventricular fractional shortening of zebrafish after MO-*slm2* or MO-Ctrl injection. **H.** Atrial fractional shortening of zebrafish after MO-*slm2* or MO-Ctrl injection. **I.** Heart rate of zebrafish after MO-*slm2* or MO-Ctrl injection. *, *P* < 0.05 (Student’s *t*-test). A, atrium; V, ventricle; HF, heart failure; WT, wild-type.

Using tblastn and homology analyses, we identified a 68% similarity between zebrafish and human SLM2 proteins, both containing the KH RNA-binding domain and the splice-mediating tyrosine (Y) residue (Figure 2A). We performed a knockdown of *slm2* in zebrafish by injection of a specific splice-site targeting morpholino-modified antisense oligonucleotide (MO) against *slm2* mRNA (MO-*slm2*). The effectiveness of MO-*slm2* was validated by a PCR analyzing the exon–intron boundaries targeted by MO-*slm2*, followed by Sanger sequencing (Figure 2B and C). The skipping of exon 4 leads to a shortened mRNA with consecutive frameshift. Zebrafish treated with MO-*slm2* predominantly exhibited a curly tail and severe abnormal cardiac morphology compared to those treated with MO-Ctrl (Figure 2D and E). The heart was stretched due to severe pericardial effusion, while the atrioventricular (AV) channel was poorly defined and blood was regurgitated through the rudimentary developed mitral valve. In addition, zebrafish with *slm2* knockdown showed a higher rate of heart failure than those treated with MO-Ctrl (Mo-*slm2 n* = 210 and Mo-Ctrl *n* = 355, respectively) (Figure 2F). The heart function was impaired after *slm2* knockdown, as measured by the reduced ventricular fractional shortening (Figure 2G), whereas atrial fractional shortening (Figure 2H) and heart rate were not changed (Figure 2I).

### Elevation of SLM2 expression alters calcium cycling in human cardiomyocytes

**Figure 3:**
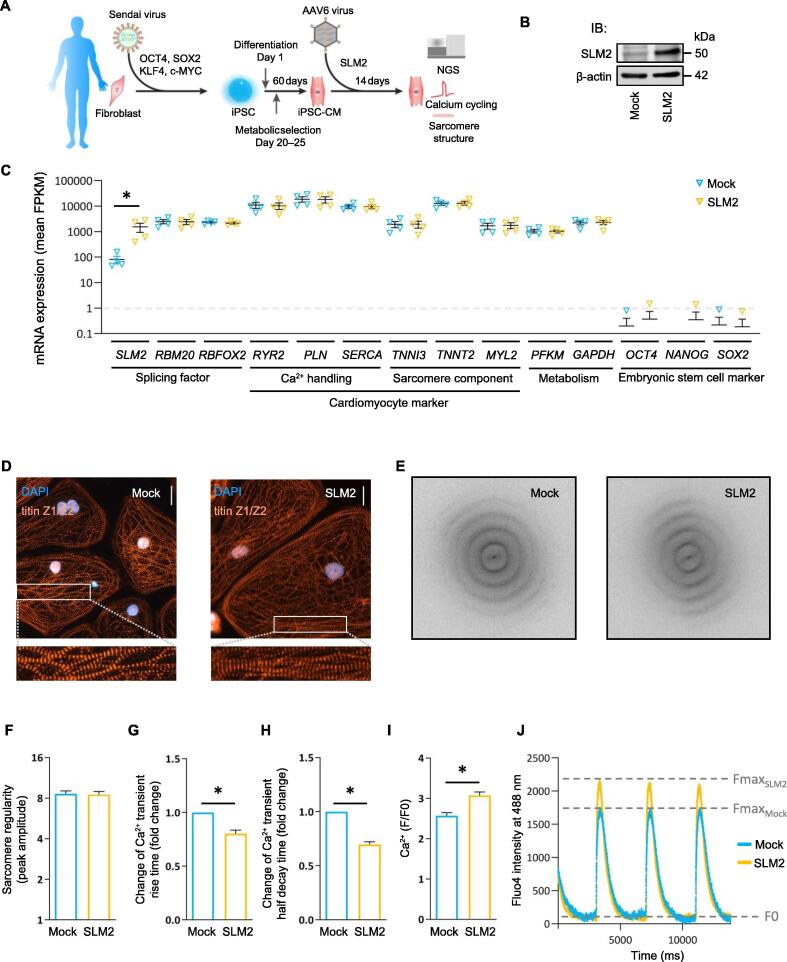
**SLM2-dependent effects in human iPSC-CMs** **A.** Diagram showing the generation of iPSC-CMs. Fibroblasts of human healthy donors were reprogrammed using non-integrating Sendai virus to generate iPSC clones. iPSC-CMs were then generated by Wnt modulation and metabolic selection. **B.** IB showing AAV6-mediated overexpression of SLM2 in iPSC-CMs. β-actin was used as a loading control. Mock indicates iPSC-CMs transfected with control AAV6 virus. **C.** Gene expression after overexpression of *SLM2* by transduction with AAV6 virus (*n* = 4) analyzed by NGS. **D.** Immunofluorescence assay of the sarcomeric protein titin Z1/Z2. Scale bar, 50 µm. **E.** and **F.** Determination of sarcomere regularity using FFT. Four cardiac differentiation experiments for one cell line were used, and single iPSC-CMs were analyzed (Mock: *n* = 74 iPSC-CMs; SLM2: *n* = 85 iPSC-CMs). **G.** Change of Ca^2+^ transient rise time. **H.** Change of Ca^2+^ half decay time. **I.** and **J.** [Ca^2+^]i (F/F0) after overexpression of *SLM2* in iPSC-CMs (Mock, *n* = 66; *SLM2*, *n* = 66). Data are presented as mean ± SEM. Statistical differences were calculated using Student’s *t*-test (*, *P* < 0.05). iPSC, induced pluripotent stem cell; iPSC-CM, iPSC-derived cardiomyocyte; AAV6, adeno-associated virus serotype 6; NGS, next-generation sequencing; FFT, Fast Fourier Transform.

We next investigated the role of SLM2 in induced pluripotent stem cell-derived cardiomyocytes (iPSC-CMs), which were derived from human healthy donors and generated by Wnt modulation and metabolic selection (Figure 3A). By gene transfer with adeno-associated virus serotype 6 (AAV6) particles, we overexpressed *SLM2* in these functional human cardiomyocytes (Figure 3B and C). iPSC-CMs showed high expression of cardiac structural and calcium-associated marker genes, such as *TNNI3*, *TNNT2*, *MYL2*, *RYR2*, *SERCA*, and *PLN*, whereas the expression of pluripotency genes *OCT4*, *NANOG*, and *SOX2* was below the limit of detection (Figure 3C). Also, phenotypical analysis (Figure 3D) and evaluation using Fast Fourier Transform (FFT) (Figure 3E) showed no significant changes in the sarcomere regularity after *SLM2* overexpression (Figure 3F). However, *SLM2* overexpression resulted in altered calcium cycling kinetics compared to control AAV6-transduced iPSC-CMs. We found a significantly reduced Ca^2+^ transient rise time and a significantly reduced half decay time after *SLM2* overexpression (Figure 3G and H). Therefore, the Ca^2+^ transient amplitude was significantly increased in *SLM2*-overexpressed iPSC-CMs (Figure 3I), resulting in an increase of F_max_ (Figure 3J).

### SLM2 binds differentially spliced sarcomeric transcripts in failing human hearts

**Figure 4:**
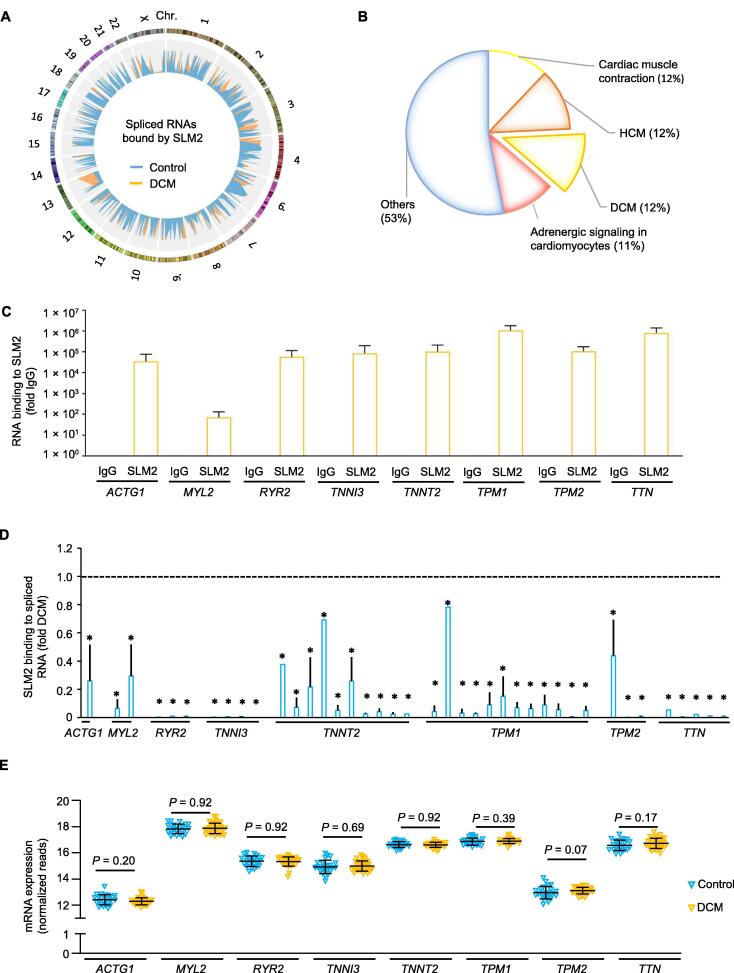
**SLM2 binding to RNAs in the failing human heart** **A.** Circos plot showing 172 SLM2-bound RNAs which exhibit significant changes in alternative splicing (indicated by PSI). SLM2 was immunoprecipitated from myocardial tissue lysates of the left ventricle from DCM patients (*n* = 3, orange) and controls (*n* = 4, blue), respectively, and bound RNAs were deep sequenced. Chr. 5 and Chr. Y were excluded due to lack of significant associations. **B.** GO analysis of the disease relevance of the 172 SLM2-bound RNAs showing differential splicing between DCM and control hearts (*P* < 0.05 indicating false discovery rate by Benjamini). **C.** Enrichment of alternatively spliced RNAs by SLM2 RIP from DCM hearts followed by sequencing. Data are presented as mean ± SD (*n* = 3 DCM samples). **D.** Alternative splicing of RNAs bound to SLM2 in hearts of DCM patients (*n* = 3) compared to controls (*n* = 4). Student’s *t*-test (*, *P* < 0.05). **E.** Total gene expression of the selected SLM2-bound RNA candidates in the hearts of DCM patients (*n* = 33) and controls (*n* = 24). Student’s *t*-test. PSI, percent spliced in; GO, Gene Ontology; RIP, RNA immunoprecipitation.

To elucidate the potential downstream effectors of SLM2 in the human heart, we performed RNA immunoprecipitation (RIP) of SLM2 followed by next-generation sequencing (RIP-seq). We precipitated endogenous SLM2 from myocardial tissue lysates of the left ventricle of DCM patients and non-diseased controls, respectively. Alternative splicing of RNAs bound to SLM2 was analyzed, being a perquisite for SLM2 enzymatic activity (Figure 4A). A total of 172 SLM2-bound RNAs were identified to be significantly differentially spliced between DCM and control hearts. These identified RNAs were then analyzed using Gene Ontology (GO) to reveal their association with human diseases. We found that 47% of the differentially spliced RNAs were associated with myocardial physiology and diseases, such as hypertrophic cardiomyopathy (HCM) and DCM (Figure 4B). Alternatively spliced RNA elements of pivotal myocardial genes, such as actin gamma 1 (*ACTG1*), myosin light chain 2 (*MYL2*), ryanodine receptor 2 (*RYR2*), troponin I3 (*TNNI3*), troponin T2 (*TNNT2*), tropomyosin 1 (*TPM1*), tropomyosin 2 (*TPM2*), and titin (*TTN*; Table 1), were enriched by SLM2 RIP in DCM samples (Figure 4C). Interestingly, several of the SLM2-enriched mRNAs were found to be alternatively spliced at multiple sites (Figure 4D), while being not changed on total gene expression level in the diseased hearts (Figure 4E).

**Table 1:** **SLM2-bound *TTN* transcripts/exons in the heart tissues of DCM patients**

**Gene**	**Exon**	**Transcript**	**SLM2-bound in DCM *vs.* control (fold change)**	***T* statistic**	***P* value**
*TTN*	89	205:ENST00000425332+ENST00000589042	18.614	−2.692	0.027
*TTN*	92	204:ENST00000425332+ENST00000589042	227.126	−2.734	0.025
*TTN*	103	210:ENST00000589042	44.288	−3.029	0.016
*TTN*	131	241:ENST00000591111+ENST00000342992+ENST00000589042+ENST00000615779	95.296	−4.139	0.003
*TTN*	139	181:ENST00000589042	104.234	−4.564	0.001

*Note*: Exons/transcripts of *TTN* mRNA which bound to SLM2 were identified in the heart tissues of DCM patients using RIP-seq. SLM2, Sam68-like mammalian protein 2; TTN, titin; DCM, dilated cardiomyopathy; RIP-seq, RNA immunoprecipitation followed by next-generation sequencing.

### SLM2 regulates alternative splicing of ***TTN*** mRNA in the myocardium of DCM patients

**Figure 5:**
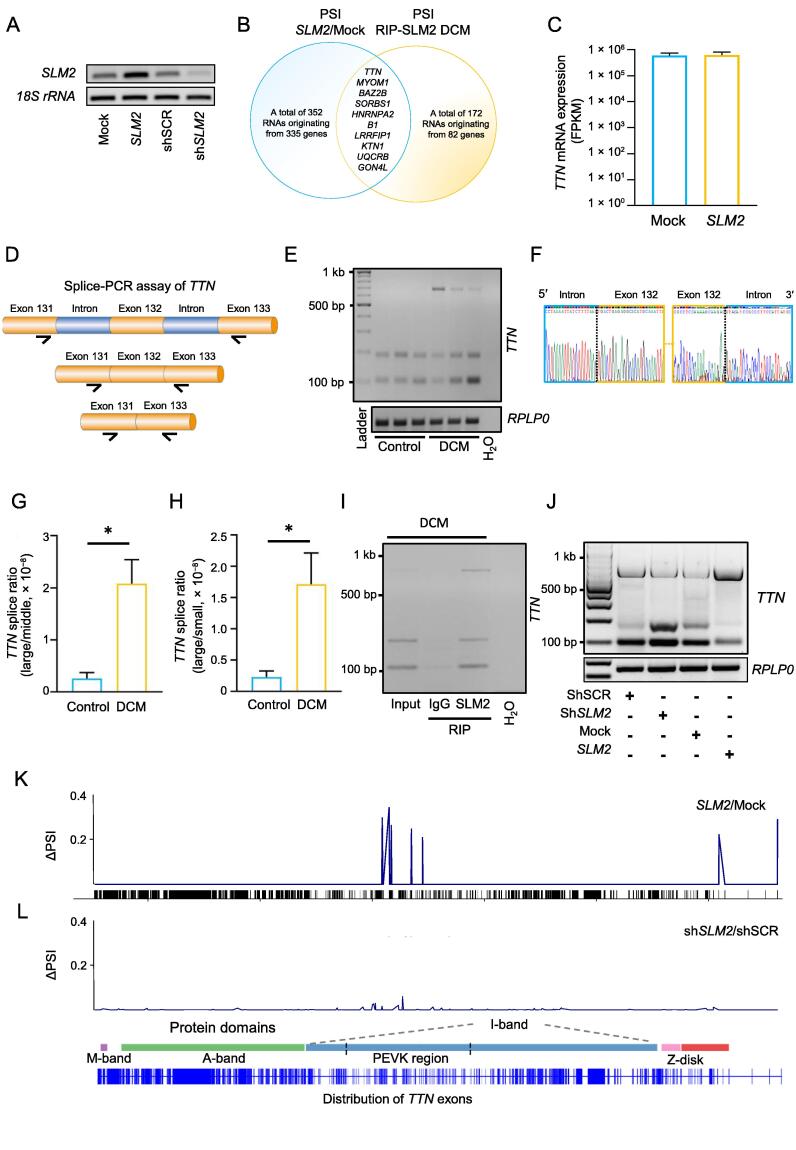
**SLM2 modulates *TTN* mRNA splicing** **A.** PCR analysis validating the increase and reduction of *SLM2* expression in human iPSC-CMs via gene transfer with AAV6. **B.** Overlapping of SLM2-bound RNAs differentially spliced in DCM hearts and RNAs differentially spliced after overexpression of *SLM2* in human iPSC-CMs. **C.***TTN* mRNA expression after overexpression of *SLM2* in human iPSC-CMs (*n* = 4). **D.** Schematic of the pre-mRNA region of *TTN* analyzed by splice-PCR. The region contains exon 131 (ENSE00003808052), exon 132 (ENSE00003803067), exon 133 (ENSE00003808806), and two intermediate introns of *TTN* pre-mRNA (ENST00000589042). Arrows indicate the predicted binding sites of the primers. **E.** Splice-PCR analyzing *TTN* mRNA splicing in DCM patients (*n* = 3) and controls (*n* = 3). **F.** Sanger sequencing of the splice-PCR products showing the exemplary exon–intron junction of the largest splice (686 bp) product. **G.** and **H.** Quantification of the splice ratios of the large *TTN* splice product (686 bp) to the middle sized *TTN* splice product (187 bp) (G) and the large *TTN* splice product (686 bp) to the small *TTN* splice product (103 bp) (H) as shown in (I). Student’s *t*-test (*, *P* < 0.05). **I.** Interaction of SLM2 with *TTN* mRNA in the myocardial lysate from a DCM patient using RIP. IgG served as an antibody control. **J.** Splice-PCR analyzing *TTN* mRNA splicing after increase and reduction of *SLM2* expression in human iPSC-CMs. **K.** and **L.** Percentage “spliced-in” compared to the whole *TTN* gene expression (mean PSI of *n* = 4).

PCR analysis validated the increase and reduction of *SLM2* expression in human iPSC-CMs via gene transfer with AAV6 (Figure 5A). We next determined the deferentially spliced RNAs in iPSC-CMs after *SLM2* overexpression, and combined those with differentially spliced SLM2-bound RNAs identified in the DCM hearts. Interestingly, *TTN* was differentially spliced in iPSC-CMs after *SLM2* overexpression (Figure 5B), but did not exhibit changes on total gene expression level (Figure 5C). A splice-PCR assay was designed to validate the alternative splicing events of *TTN* mRNA identified by genome-wide SLM2 RIP-seq in DCM hearts (Figure 5D). We found that two introns in the region of exons 131–133 were retained in the *TTN* mRNA of DCM patients compared to controls, which directly reflects the status change in the *in vivo* situation of humans (Figure 5E). The PCR products were isolated and validated using Sanger sequencing, showing exemplary exon–intron junction of the largest isoform (686 bp) (Figure 5F). Quantitative analysis showed the predominance of the largest, intron-containing splice variant compared to the small and middle *TTN* splice variants in DCM hearts, compared to controls (Figure 5G and H). To validate the proposed interaction of SLM2 with this differentially spliced *TTN* mRNA region, RIP followed by splice-PCR was performed in the myocardial lysate of a DCM patient. The results showed that the amount of the largest, intron-containing isoform was increased after enrichment for SLM2 compared to IgG control (Figure 5I). We next analyzed the SLM2-mediated effect on *TTN* mRNA splicing in iPSC-CMs after increase or reduction of *SLM2* expression (Figure 5J–L). We found that overexpression of *SLM2* in iPSC-CMs results in increased intron retention in the proline-, glutamate-, valine-, and lysine-rich (PEVK) domain of titin, as shown by increase in both the amount of the largest isoform and the Δ percent spliced index (ΔPSI) (Figure 5J and K). These effects resemble the effects seen in DCM tissue samples (*i.e.*, more intron retention of *TTN*). Accordingly, reduction of *SLM2* expression resulted in the opposite splicing behavior, *i.e.*, less intron retention of *TTN* (Figure 5J and L).

### SLM2 promotes intron retention and prevents exon exclusion of ***TTN*** mRNA

**Figure 6:**
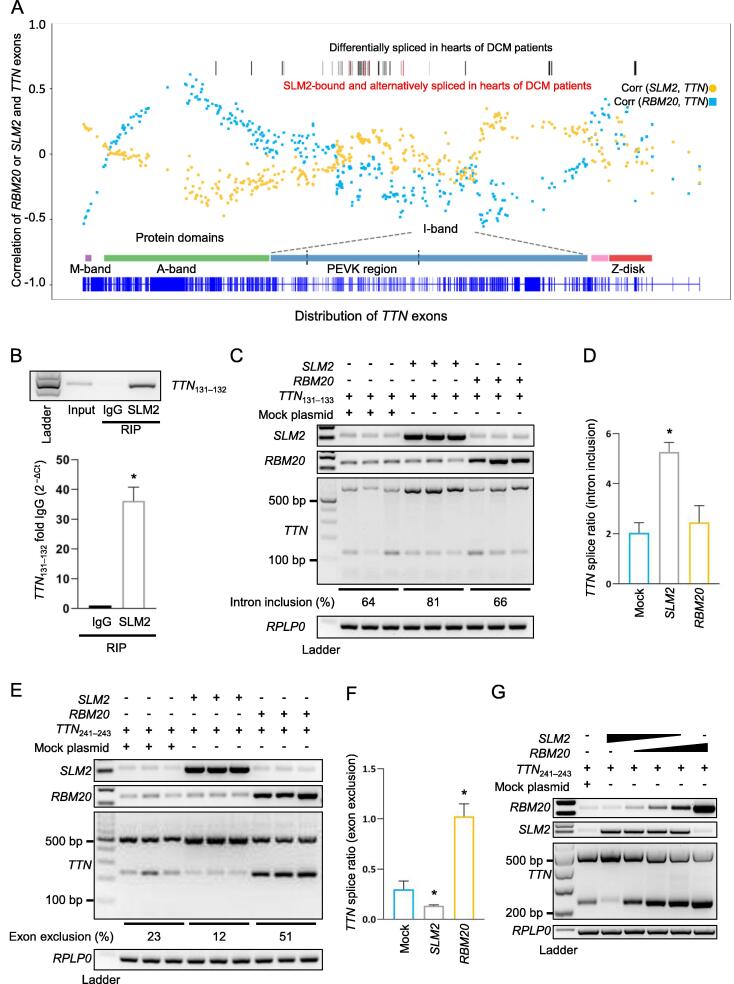
**SLM2 promotes intron retention and prevents exon exclusion in *TTN* mRNA opposing the role of RBM20** **A.** Correlation coefficients of *TTN* exon expression to RNA expression of *RBM20* (blue) and *SLM2* (yellow) in LV biopsies analyzed by RNA-seq in DCM patients (*n* = 56). **B.** Binding of SLM2 to *TTN* exons 131–332 verified by RIP followed by RT-PCR. *n* = 3. **C.** RT-PCR showing *TTN* mRNA splicing after transfection of the *TTN*_131–133_ minigene along with *SLM2* or *RBM20*. **D.** Quantification of *TTN* mRNA splice ratios as shown in (C). *n* = 3. **E.** RT-PCR showing *TTN* mRNA splicing after transfection of the *TTN*_241–243_ minigene along with *SLM2* or *RBM20*. **F.** Quantification of *TTN* mRNA splice ratios as shown in (E). *n* = 3. **G.** RT-PCR showing *TTN* mRNA splicing after transfection of the *TTN*_241–243_ minigene along with increasing *RBM20* and decreasing *SLM2*. *, *P* < 0.05 (Student’s *t*-test). LV, left-ventricular.

The differentially spliced exons of *TTN* mRNA bound to SLM2 protein in hearts from DCM patients were compared with all alternative splicing events in the *TTN* mRNA in DCM. This analysis revealed an enriched SLM2 interaction with *TTN* exons encoding the PEVK domain. The PEVK domain acts as an entropic spring mediating flexibility and contractile properties of the heart muscle. We analyzed the correlation of *SLM2* expression with the expression of each exon of *TTN* and compared it to the correlation of *RBM20* expression with the expression of each exon of *TTN*. The *TTN* exons encoding the PEVK region had the highest correlation coefficients with *SLM2* expression, whereas the correlation coefficients of *TTN* exons with the gene expression of the well-known splicing regulator RBM20 were low in this region (Figure 6A). To study the molecular basis of SLM2-dependent *TTN* intron inclusion, which we found by SLM2-RIP-seq in DCM patients, we designed a minigene construct consisting of human *TTN* exons 131–133 (named *TTN*_131–133_). This part of titin’s PEVK region was transferred as an exon/intron cassette into a minigene expressed under the control of a CMV promoter. RIP showed a significant binding of SLM2 to this *TTN* mRNA region (Figure 6B). Transient transfection of *TTN*_131–133_ with addition of *SLM2* resembled the effect seen in DCM tissue samples, resulting in more intron retention and longer isoform expression of *TTN*, whereas RBM20 had no effect on the splicing of this region (Figure 6C and D). Next, we tested the effect of SLM2 on splicing of the human *TTN* exons 241–243, which are part of titin’s I-band region and recently have been shown to be regulated by RBM20 [26]. Consistently, we found RBM20 to mediate exon exclusion. Interestingly, SLM2 had the opposing effect, resulting in significant higher exon inclusion (Figure 6E and F). The effects of RBM20 and SLM2 on *TTN* exon exclusion/inclusion were validated by increasing *RBM20* level, while at the same time decreasing *SLM2* level (Figure 6G).

### Identification of the SLM2-binding motifs in ***TTN*** mRNA

**Figure 7:**
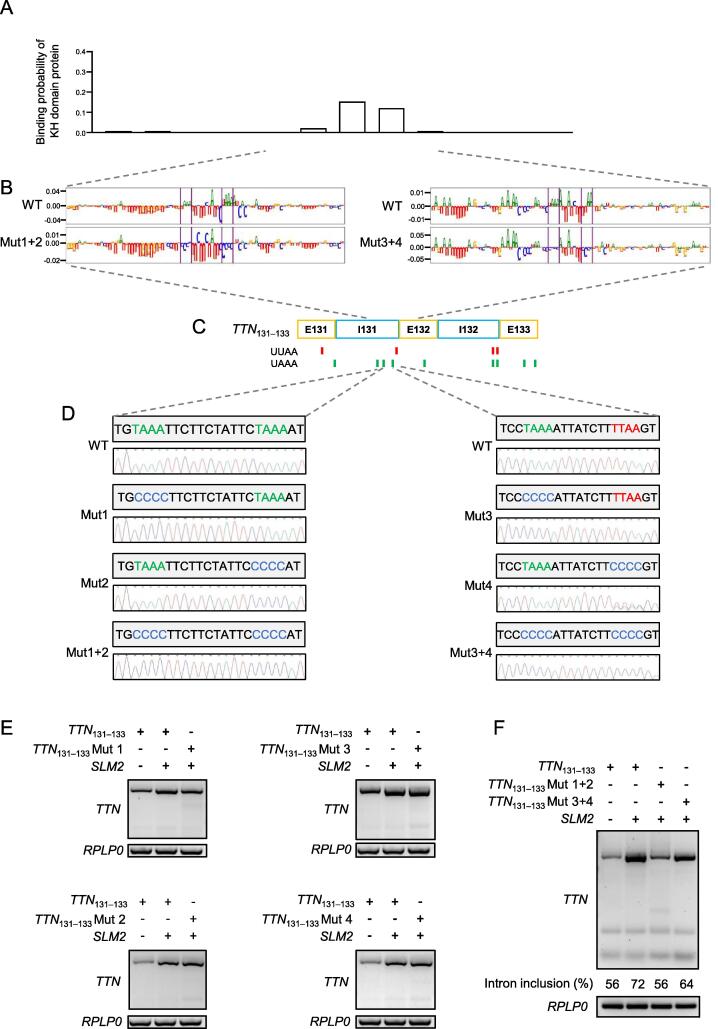
**Identification of the SLM2-binding motifs in *TTN* mRNA** **A.** Binding probability of SLM2 to *TTN* intron 131 predicted from CLIP datasets using the DeepRiPe machine learning model. **B.** Potential SLM2-binding UWAA repeat motifs in intron 131 of *TTN* mRNA. **C.** Occurrence of UWAA motifs in the *TTN* exons 131–133. **D.** Sanger sequencing showing individual (Mut1, Mut2, Mut3, and Mut4) and double (Mut1+2 and Mut3+4) mutations of the identified UWAA motifs in intron 131 in the *TTN*_131–133_ minigene. **E.** and **F.** Analysis of *TTN* mRNA splicing using splice-PCR targeting exons 131–132 after transfection of HEK293 cell with the WT *TTN*_131–133_ minigene or *TTN*_131–133_ minigene with individually (E) or doubly (F) mutated UWAA motifs along with a plasmid expressing human *SLM2*. CLIP, crosslinking and immunoprecipitation; DeepRiPe, Deep RBP binding preference.

We used Deep RBP binding preference (DeepRiPe) [27], an interpretable machine learning model, to characterize SLM2 binding preferences from available crosslinking and immunoprecipitation (CLIP) datasets including ENCODE eCLIP data. Using the genome sequence of the *TTN* exons 131–133 as an input to the model, we detected potential binding of SLM2 to nucleotides located in intron 131 of *TTN* (Figure 7A), and identified an intron-enriched UWAA repeat motif (Figure 7B and C). Indeed, SLM2 has been shown to act on the UWAA repeats upstream of an alternative exon of *Neurexin2* mRNA [28]. Therefore, we mutated the UWAA motifs individually (Mut1, Mut2, Mut3, and Mut4) and doubly (Mut1+2 and Mut3+4) (Figure 7D) to investigate the importance of the UWAA motifs. The wild-type *TTN*_131–133_ minigene or *TTN*_131–133_ minigene with mutated UWAA motifs was transfected into HEK293 cells together with a plasmid expressing human *SLM2*. We found that Mut1 had a modest impact on intron inclusion (Figure 7E). In line, the doubly mutated UWAA motifs (Mut1+2) resulted in decreased intron inclusion, whereas Mut3+4 showed only a modest effect on splicing (Figure 7F). Together, this result indicates that SLM2 recognizes specific splice motifs, *e.g.*, in *TTN*, to regulate mRNA processing in the heart.

## Discussion

Alternative splicing in the human heart is important to adapt to changing physiological demands, aging, and disease. This study demonstrates that the splicing regulator SLM2 is up-regulated in the failing myocardium of DCM patients and is actively involved in cardiac mRNA splicing of multiple pivotal cardiac genes including *TTN*. Importantly, loss  or overexpression of SLM2 results in hallmarks of heart failure and pinpoints to its functional role in the heart.

Cellular functional diversity is regulated by alternative splicing and critical for adaptation in cardiac development and heart failure. Nevertheless, we are only beginning to understand various factors contributing to this important adaptive mechanism. Tissue-specific splicing regulators are classified based on their domains [29]. Several splicing factors have been linked to cardiac pathophysiology; however, the majority were verified in non-human models [6], [9], [10], [18], [30], [31], [32], [33], [34], [35]. Using a genome-wide approach in humans by combining expressional regulation of mRBPs in the diseased myocardium and a score of RBP tissue enrichment as an evolutionary surrogate of functional relevance, our experiments identify the splicing factor SLM2 of the STAR family that was enriched in myocardial tissue and up-regulated in the failing human heart. Expression of the splicing factor gene *RBM20* is tightly regulated during embryogenic development of the cardiac system and has been implicated in the adaptation of the sarcomere to the changing mechanical needs during the perinatal period [9]. Importantly, we found that SLM2 but not RBM20 was regulated in the setting of heart failure in humans. This might point to diverging roles of SLM2 and RBM20 in adaptation to myocardial stress.

Targets of cardiac splicing regulators have been successively identified and are diverse but frequently conserved across species. SLM2 has previously been shown to regulate the splicing of the transcripts of polymorphic synaptic receptors neurexin1, neurexin2, and tomosyn-2 in neurons [36], [37], [38], [39]. The STAR domain, which comprises a KH domain and flanking regions and mediates protein–protein and protein–RNA interactions, was found to be responsible for establishing differential splicing repression of *Neurexin2* mRNA, while the interaction was dependent on UWAA-rich sequences flanking the alternative exons on RNA level [28]. As shown here, RBM20 and SLM2 share several of their targets in the heart, including *TTN*, which are important parts of the heart failure phenotype observed in humans with mutated *RBM20* [20], [40]. The resulting exon and intron composition changes, however, might be different for these two splicing regulators.

The data presented here are derived from human heart tissues and underline the regulation of SLM2 during DCM. To further underline the human relevance, we constructed adenoviruses overexpressing *SLM2* in wild-type human iPSC-CMs. As shown, *SLM2* has profound effects on calcium homeostasis and kinetics, which is a hallmark of DCM. *SLM2* overexpression did not result in severe malformations of the sarcomeres, which we did not expect since SLM2 was elevated in DCM patients of different disease severities and our histological analysis did not show alterations pointing toward this direction.

Mutations in genes encoding splicing factors often result in severe disease phenotypes in humans, as observed in cardiomyopathies which are often associated with mutations in *RBM20* [41], [42], [43]. Using the Genome Aggregation Database (gnomAD) resource, *SLM2* has a constraint score observed/expected (o/e) of 0.73 for missense variants and 0.22 for loss-of-function variants, while synonymous variants have a score of 0.99. These scores are highly similar to those of *RBM20* and thereby indicate intolerance for deleterious mutations during evolution. This is also reflected by the total allele number of loss-of-function and missense variants for *SLM2* in the non-Finnish European haplotype cohort in gnomAD, *i.e.*, only 157 of 64,542 alleles (ratio 0.0024) are affected by such a variant. We found a similar variant frequency (ratio 0.0017) in an available cohort of DCM patients (*n* = 580), *i.e.*, only one variant at a predicted splice-donor site (ENST00000355849.10:c.207+1G>T) in one DCM patient; however, we did not find any missense variant in *SLM2*. The rare occurrence of such mutations might be explained by the severe functional consequence, *i.e.*, a multitude of mRNA targets may be misspliced by a mutated splicing factor; however, mutations in a gene encoding a structural protein such as titin result in a more favorable clinical course of cardiomyopathy.

Using RIP, we observed interactions of SLM2 with several mRNAs encoding proteins of the contractile apparatus of the myocardium such as myosin light chain 2, troponin I and T, tropomyosin 1 and 2, and titin. In the failing human myocardium, we found that SLM2 binds to alternative mRNA components encoding the PEVK domain of titin, resulting in intron inclusion, which was also validated in a cell culture model. While we also identified alternative exon usage, intron inclusion might be the more interesting mechanism in light of the disease-associated regulation of SLM2. A recent study has shown intron retention to be a critical regulator of gene programs, especially in response to stress and disease [44]. Various newer studies showed how intron retention regulates RNA stability, nonsense-mediated mRNA decay, protein isoform expression, and translation efficiency [45]. Furthermore, rapid induction of protein translation via post-transcriptional splicing of mRNAs containing retained introns might be crucial for a stressed cell to counteract any further damage. Mechanistically, the results from our minigene experiments revealed that elevated *SLM2* level resulted in enhanced intron inclusion within the mRNA region encoding the PEVK domain of titin, underlining our hypothesis. Interestingly, elevated *SLM2* level also resulted in exon inclusion in another part of the I-band region of titin, which was not spotted by the RIP experiments. Here, SLM2 counteracted the repressor activity of RBM20 on the splicing of the exons 241–243. Hence, it should be further explored if *SLM2* overexpression can also counteract the detrimental loss of *RBM20* function.

In summary, we identified SLM2 as an important splicing factor in human cardiomyocytes, which is elevated in the failing myocardium. The changes in the SLM2-mediated splicing of the mRNA regions encoding the PEVK domain and another part of the I-band region of titin, which act as entropic springs mediating flexibility and contractile properties of the heart muscle, suggest that the heart failure-associated up-regulation of *SLM2* might be a compensatory mechanism to adapt to the increased wall stress and the hypertrophic response of the heart [46].

## Materials and methods

### Patient inclusion and sampling

The diagnosis of DCM was confirmed after excluding coronary artery disease (determined by coronary angiography), valvular heart disease (determined by cardiac magnetic resonance imaging and echocardiography), and myocarditis/inflammatory DCM (determined by histopathology) [24]. Patients with a history of uncontrolled hypertension, regular alcohol consumption, illicit drug use, or cardiotoxic chemotherapy were also excluded. To include the clinical continuum of systolic heart failure, early but symptomatic disease stages [left-ventricular (LV) ejection fraction between 45% and 55%] were included. Patients routinely biopsied after heart transplantation served as controls for mRNA-seq-based expression detection. Importantly, only patients with normal cardiac function and without transplant rejection were included as controls.

Patients with ICM (defined by decreased systolic function and absence of coronary artery disease) were also included for qPCR expression analysis.

### RNA analysis in human heart tissue

Human heart tissue (100 mg) was washed with ice-cold DPBS, and then lysed in 700 µl QIAzol (Catalog No. 79306, Qiagen, Hilden, Germany) in a Precellys 24 Tissue Homogenizer (Bertin Instruments, Rockville, MD) with 5000 r/min at 4 °C until fully lysed. Total RNA was isolated using the miRNeasy Kit (Catalog No. 217004, Qiagen) with additional DNase I (Catalog No. 79254, Qiagen) digestion according to the manufacturer’s protocol. Then, 1 μg of RNA from each sample was reverse-transcribed into single-strand cDNA using random hexamer primers (10 min at 25 °C, 15 min at 42 °C, 5 min at 99 °C) by MMLV Reverse Transcriptase (Catalog No. N8080018, Invitrogen, Carlsbad, CA).

For qPCR, cDNA was amplified using Fast SYBR Green Mastermix (Catalog No. 4385612, Life Technologies, Carlsbad, CA) on a VIAA7 qPCR System (Life Technologies) with intron-spanning primer pairs (Table S1). Expression levels of mRNAs were normalized to *RPLP0* which served as a reference gene using the 2^–ΔCt^ method [47].

### RIP

Human heart tissue (100 mg) was washed with ice-cold DPBS, and then lysed in 300 µl RIP lysis buffer from Magna RIP Kit (Merck KGaA, Darmstadt, Germany) in a Precellys 24 Tissue Homogenizer (Bertin Instruments) with 5000 r/min at 4 °C until fully lysed. Ribonucleic complexes were precipitated using 5 µg of SLM2 antibody (Catalog No. A303-191A, Bethyl, Montgomery, TX) and 5 µg of rabbit isotype IgG control (Catalog No. PP64B, Merck KGaA, Darmstadt, Germany). The Magna RIP RNA-Binding Immunoprecipitation Kit (Catalog No. 17–700, Merck KGaA) was used according to the manufacturer’s instructions [48]. Precipitated RNA was eluted in 25 µl nuclease-free H_2_O, converted into cDNA, and then analyzed using PCR or next-generation sequencing.

### IB analysis

Protein was isolated from human LV biopsies in lysis buffer using a Precellys 24 Tissue Homogenizer (Bertin Instruments) with 5000 r/min at 4 °C until fully lysed. Lysates were used for SDS-PAGE followed by protein transfer to membrane. Membranes were incubated with either SLM2 antibody (Catalog No. A303-192A, Bethyl) or the antibody targeting sarcomeric actin as a loading control (Catalog No. A 2172, Sigma Aldrich, St. Louis, MI).

### Calculation of HSS

Definition of 1542 RBP genes was obtained from recent publication [23]. The mean expression of these 1542 genes was determined in 53 human tissues (Table S2) and was extracted from the Genotype-Tissue Expression (GTEx) database (https://www.gtexportal.org/home/). Specifically, the GTEx_Analysis_v6_RNA-seq_RNA-SeQCv1.1.8_gene_rpkm file was downloaded from the GTEx database and used for extracting the Reads Per Kilobase per Million mapped reads (RPKM) values of the RBP genes. A total of 31 genes was missing from the GTEx file and therefore these were not included for further analysis. The median RPKM value of the averaged RPKM value of each gene across 53 tissues is 8.95 and the median standard deviation is 3.91. The RBP genes that have mRNAs as their consensus targets were used for further analysis. HSS was defined as the ratio between the mean expression in non-heart tissues to the expression in the heart (left ventricle) tissue, for each gene. This value was averaged across various tissues and sorted in the descending order. A high value of the ratio corresponds to a high degree of heart specificity. Heart specificity was also evaluated in the same way using the expression values of atrial-appendage tissue to validate the results.

### Analysis of RBPs in the heart of DCM patients

In addition to data from the GTEx database, RNA-seq data from a cohort of 33 DCM patients and 24 controls were analyzed [49]. First, samples were subjected to a quality check using the tool FastQC (v0.11.8). The human reads were then aligned to the GRCh38 assembly. Specifically, the FASTA files were downloaded from the Ensemble FTP and the ‘hisat2-build’ command from HISAT2-2.1.0 was used to create an index for the genomes. The RNA-seq data were aligned to the index using the ‘hisat2’ command. The alignments stored in BAM format files were sorted using Sambamba_v0.6.6 followed by assembly of transcripts using StringTie-1.3.3b, to produce GTF files with gene and transcript abundances. StringTie was then run with the ‘merge’ option to produce merged GTF files. StringTie was also run with the ‘-e’ option, and the resulting GTF files were used as an input to the prepDE.py script provided by the StringTie authors to extract read count information at the gene and transcript levels as a matrix. The Fragments Per Kilobase per Million mapped reads (FPKM) values for the given genes were then extracted. The median gene expression of the averaged value of the genes across all samples is 3.00 and the median standard deviation is 1.85. In case of the pull-down samples, the matrix with the read count data was used as an input to the DESeq2 R package to obtain normalized counts at the gene and transcript levels. The tool featureCounts (v1.6.2) was used to summarize expression at the exon level, and the raw counts generated by featureCounts were normalized using the DESeq2 package. Differential expression was tested using Student’s *t*-test and considered significantly changed when *P* < 0.05.

### Calculating PSI

A recently described pipeline was followed to calculate the PSI of the exonic regions in the human genome [50]. The GTF file corresponding to human (Homo_sapiens.GRCh38.90.gtf) was obtained from Ensemble. The dexseq_prepare_annotation.py script was used to extract the exonic parts of the genome. Regtools ‘junctions extract’ command was used to create a junctions.bed file. The commands used for creating the PSI file as described in [50] were then used sequentially. The exonic regions were sorted according to their gene IDs. Python SciPy library (https://www.scipy.org) was used to perform *t*-test to determine any significant differences between the PSI values of the control and DCM after SLM2 pull-down.

### Cell culture and generation of iPSC-CMs

Fibroblasts or peripheral blood cells of human healthy donors (*n* = 4) were reprogrammed using non-integrating Sendai virus to generate high-quality iPSC clones, which were expanded as described [51]. iPSC-CMs were differentiated using Wnt modulation and metabolic selection deleting less differentiated cardiomyocytes as described [51]. Fully differentiated cardiomyocytes did not express pluripotency markers such as *OCT4*, *NANOG*, and *SOX2*, but exhibited high expression of genes encoding cardiac troponins and cardiac myosin chains such as *TNNI3*, *TNNT2*, and *MYL2*. The iPSC-CMs showed sarcomeric structures and cardiac calcium cycling.

### Gene expression manipulation by AAV6

Full differentiated iPSC-CMs were transduced with AAV6 particles. A multiplicity of infection (MOI) of 1 × 10^3^ was used for the AAV6-mock (AAV6-GFP; Catalog No. 7008, Vector Biolabs, Malvern, PA) and AAV-*SLM2*-overexpression (Catalog No. AAV6-h-KHDRBS3, Vector Biolabs). MOI of 1 × 10^5^ was used for the control AAV6-shscrambled (AAV6-GFP-U6-shRNA; Catalog No. 7043, Vector Biolabs) and the AAV-*SLM2*-shRNA (Catalog No. shAAV6-212876, Vector Biolabs). iPSC-CMs were incubated with AAVs for 3 days and used after 11 more days of cultivation.

### Calcium imaging

Intracellular calcium (Ca^2+^) transients were imaged in iPSC-CMs at days 70–90 of cardiac differentiation as described [51]. Briefly, 250,000 iPSC-CMs were plated onto Geltrex-coated 25-mm round coverslips. After 5–7 days, iPSC-CMs were transduced with AAV6 viruses. For imaging, iPSC-CMs were incubated with 2.5 µM of a Ca^2+^‐fluorescent probe RHOD-2 (Catalog No. R1244, Invitrogen) for 30 min in Tyrode’s solution containing 140 mM NaCl, 5.4 mM KCl, 1 mM MgCl_2_, 10 mM glucose, 1.8 mM CaCl_2_, and 10 mM HEPES (pH 7.4). iPSC-CMs were paced at 0.25 Hz and Ca^2+^ transients were recorded at room temperature with a Zeiss LSM Axio Observer Z1 confocal microscope with the following settings: Laser 516 nm with 0.4%–1.8 %, line scan mode with 20,000 cycles and zoom 3. For statistical analysis, the fold change was calculated by dividing the *SLM2* overexpression by the mean of the basal mock control values.

### Measurement of sarcomere regularity

A total of 30,000 iPSC-CMs were plated onto Geltrex-coated 20-mm round coverslips and transduced with AAV6 viruses. Images of single cells stained for Z1/Z2-titin (Catalog No. 69151, Myomedix, Neckargemünd, Germany) were processed by a customized routine in ImageJ to quantify sarcomere regularity. Briefly, images were processed with the plugin “Tubeness” and 2D FFT, which results in a 1D representative radial profile. This radial profile was blotted using Labchart and the amplitude of the first-order peak was analyzed as a representative parameter for sarcomeric regularity.

### Zebrafish handling

Zebrafish (*Danio rerio*) maintenance, mating, and spawning were performed as previously described [52]. The wild-type zebrafish AB strain (Catalog No. 1175, European Zebrafish Research Center, Karlsruhe, Germany) was used. Maintenance took place at constant temperature of water and the surrounding at 28.5 °C and a defined light/dark cycle of 11 h/13 h. The aquarium system was manufactured by Schwarz (Göttingen, Germany).

### MO microinjection

To analyze the function of *SLM2*, a knockdown experiment was performed by injection of MO-*slm2*-e4i4 (5′-GTTTGAGCTACTCACATATTCTCCA-3′). Two transcript variants were annotated for *slm2* (also known as *KHDRBS3*; Ensembl: ENSDARG00000101020) in zebrafish with the following IDs for proteins (Uniprot: A0A2R8QPV6 and A0A0R4IJS6) and RNAs (Ensemble: ENSDART00000183236.1 and ENSDART00000170533.2). The MO to knock down *slm2* in zebrafish was therefore designed to target both known transcript variants. The standard control MO (MO-Ctrl; 5′-CCTCTTACCTCAGTTACAATTTATA-3′) was used a control. Both MOs were provided by Gene Tools (Philomath, OR), and were diluted in 200 mM KCl (pH = 7.5) to a 1 mM stock solution.

Injection of MOs was performed with the injection device Femtojet (Eppendorf, Hamburg, Germany). An injection ramp made of 3% agarose (in E3-buffer) was produced with an injection mold TU-1 (Adaptive Science Tools, Worcester, MA), which contained asymmetric gullies (5 cm length × 3 mm width). The injection needles were made of 1-mm glass capillaries (World Precision Instruments, Sarasota, FL) with a Narishige PC-10 device (Tokyo, Japan) with two heating steps of 72 °C and 67 °C. Injection was performed at 1–2-cell stages of the embryos. The embryos were placed into the asymmetric gullies and the MOs were injected into the zebrafish yolk with a capillary pressure of 15 hPa and an injection duration of 0.1 s. The injection pressure was adjusted to the injection needle. MO-*slm2* was injected at a concentration of 500 μM (diluted with 200 mM KCl), and MO-Ctrl was injected at the same concentration. After microinjection, the embryos were transferred into a petri dish containing E3-buffer and incubated at 28.5 °C for 6 h. Living embryos were transferred into fresh E3-buffer and at 20–22 h post fertilization 1× PTU was added to suppress pigmentogenesis. For validation of MO efficiency, a 320-bp sequence (exon skipping resulting in a 120-bp product instead) on exon 4 of the *slm2* cDNA was analyzed via PCR using the primer zKHDRBS3-i3e4+e4i4-F1 (5′-TTGACGTACTTGAATGGCGG-3′) and zKHDRBS3-i3e4+e4i4-R1 (5′-CTAGCCCTGGTCATCGTAGG-3′).

### Zebrafish analysis

Living embryos were transferred into 2.5% methylcellulose (in E3-buffer) onto an object slide and image documentation was done with the bright-field stereomicroscope LEICA MZFLIII (Wetzlar, Germany) using the digital LEICA DFC310 FX camera, with the cold-light source KL 1500 LCD (Schott, Mainz, Germany). Video documentation for the beating hearts was performed using the LEICA DM IRB microscope with a magnification of 1.6×, a LEICA 506,059 objective, and a LEICA DFC360 FX camera (MCU II, Kappa GmbH, Germany). Images were processed with ImageJ 1.50i (National Institutes of Health, MD). Heart rate was calculated as heart beats per min. The “Zebrafs” program was used to analyze the percentage of ventricular and arterial fractional shortening.

### SLM2-binding motif prediction

The recently published method called DeepRiPe was used to investigate the impacts of mutations on SLM2 binding events [27]. DeepRiPe uses interpretable machine learning to characterize RBP binding preferences from available CLIP datasets including ENCODE eCLIP data. The model is trained on RBPs including KHDRBS1, a protein in the same family of SLM2 (KHDRBS3) with a similar motif, to study the effects of Mut1-2 and Mut3-4 on SLM2 binding. The genome sequences centered on Mut1-2 and Mut3-4 and the corresponding wild-type sequences were used as an input for the model. Using an interpretation approach for deep neural networks, we assigned a score to each nucleotide in the input sequence that represents how important the nucleotide is for making the prediction [27]. By visualizing these scores as sequence logos for all nucleotides in the input sequence, we made an attribution map for wild-type and mutant sequences was obtained.

### Minigene splicing assay

HEK293 cells were seeded in a 6-well plate and then transfected using polyethylenimine to introduce plasmids containing the *TTN* minigene spanning the exonic region 131–133 or 241–243 along with *SLM2* or *RBM20* expressing plasmid or control plasmid [53]. Predicted SLM2-binding motifs in intron 131 of the *TTN*_131–133_ minigene were mutated using Q5 Site-Directed Mutagenesis Kit (New England Biolabs, Ipswich, MA) followed by Sanger sequencing for validation. After 48 h, RNA was reverse-transcribed and analyzed using splice-PCR.

## Ethical statement

Patients investigated in the present study were from the local biobank approved by the ethics committee (Application No. S-390/2011) and the medical faculty of Heidelberg University (Heidelberg, Germany) as described earlier [54]. The study was conducted according to the principles outlined in the Declaration of Helsinki. The participants of the center-wide biobank received verbal and written information about the purpose of the investigations. All participants have given written informed consent to allow for molecular analysis. Experiments including *Danio rerio* were performed under institutional approval (Regierungspräsidium Karlsruhe, Germany, Application No. T52/17), which conform to the guide for the care and use of laboratory animals published by The US National Institute of Health (NIH Publication No. 85-23, revised 1996). Controls in qPCR experiments have been used according to the protected health information (45C.F.R. 164.514 e2; Bioserve GmbH, Mainz, Germany) and the BCI informed consent F-641-5 (Biochain, Newark, CA).

## Data availability

Data from SLM2 RIP-seq and PSI analysis can be accessed at https://ccb-web.cs.uni-saarland.de/cms.

## CRediT author statement

**Jes-Niels Boeckel:** Conceptualization, Methodology, Formal analysis, Writing - review & editing, Investigation. **Maximilian Möbius-Winkler:** Investigation, Formal analysis, Methodology. **Marion Müller:** Investigation, Writing - original draft, Writing - review & editing, Formal analysis, Methodology. **Sabine Rebs:** Investigation, Formal analysis, Methodology. **Nicole Eger:** Investigation, Methodology. **Laura Schoppe:** Investigation, Methodology. **Rewati Tappu:** Formal analysis, Software. **Karoline E. Kokot:** Investigation. **Jasmin M. Kneuer:** Investigation. **Susanne Gaul:** Investigation. **Diana M. Bordalo:** Investigation. **Alan Lai:** Formal analysis. **Jan Haas:** Software, Formal analysis. **Mahsa Ghanbari:** Software, Formal analysis, Methodology. **Philipp Drewe-Boss:** Software. **Martin Liss:** Conceptualization. **Hugo A. Katus:** Conceptualization. **Uwe Ohler:** Conceptualization, Software. **Michael Gotthardt:** Conceptualization, Methodology, Writing - review & editing, Funding acquisition. **Ulrich Laufs:** Conceptualization, Writing - review & editing. **Katrin Streckfuss-Bömeke:** Conceptualization, Methodology, Formal analysis, Writing - review & editing, Funding acquisition. **Benjamin Meder:** Conceptualization, Methodology, Writing - review & editing, Funding acquisition. All authors have read and approved the final manuscript.

## Competing interests

The authors have declared no competing interests.

## References

[1] Will C.L., Lührmann R. (2011). Spliceosome structure and function. Cold Spring Harb Perspect Biol.

[2] Long J.C., Caceres J.F. (2009). The SR protein family of splicing factors: master regulators of gene expression. Biochem J.

[3] Manley J.L., Krainer A.R. (2010). A rational nomenclature for serine/arginine-rich protein splicing factors (SR proteins). Genes Dev.

[4] Martinez-Contreras R., Cloutier P., Shkreta L., Fisette J.F., Revil T., Chabot B. (2007). hnRNP proteins and splicing control. Adv Exp Med Biol.

[5] Iikura M., Miyamasu M., Yamaguchi M., Kawasaki H., Matsushima K., Kitaura M. (2001). Chemokine receptors in human basophils: inducible expression of functional CXCR4. J Leukoc Biol.

[6] Frese K.S., Meder B., Keller A., Just S., Haas J., Vogel B. (2015). RNA splicing regulated by RBFOX1 is essential for cardiac function in zebrafish. J Cell Sci.

[7] Darnell R.B. (2013). RNA protein interaction in neurons. Annu Rev Neurosci.

[8] Weyn-Vanhentenryck S.M, Mele A., Yan Q., Sun S., Farny N., Zhang Z. (2014). HITS-CLIP and integrative modeling define the Rbfox splicing-regulatory network linked to brain development and autism. Cell Rep.

[9] Guo W., Schafer S., Greaser M.L., Radke M.H., Liss M., Govindarajan T. (2012). *RBM20*, a gene for hereditary cardiomyopathy, regulates titin splicing. Nat Med.

[10] Yang J., Hung L.H., Licht T., Kostin S., Looso M., Khrameeva E. (2014). RBM24 is a major regulator of muscle-specific alternative splicing. Dev Cell.

[11] David C.J., Chen M., Assanah M., Canoll P., Manley J.L. (2010). HnRNP proteins controlled by c-Myc deregulate pyruvate kinase mRNA splicing in cancer. Nature.

[12] David C.J., Manley J.L. (2010). Alternative pre-mRNA splicing regulation in cancer: pathways and programs unhinged. Genes Dev.

[13] Greenway M.J., Andersen P.M., Russ C., Ennis S., Cashman S., Donaghy C. (2006). *ANG* mutations segregate with familial and “sporadic” amyotrophic lateral sclerosis. Nat Genet.

[14] Thiyagarajan N., Ferguson R., Subramanian V., Acharya K.R. (2012). Structural and molecular insights into the mechanism of action of human angiogenin-ALS variants in neurons. Nat Commun.

[15] Skorupa A., King M.A., Aparicio I.M., Dussmann H., Coughlan K., Breen B. (2012). Motoneurons secrete angiogenin to induce RNA cleavage in astroglia. J Neurosci.

[16] Gao C., Ren S., Lee JH, Qiu J., Chapski D.J., Rau C.D. (2016). RBFox1-mediated RNA splicing regulates cardiac hypertrophy and heart failure. J Clin Invest.

[17] Ma J., Lu L., Guo W., Ren J., Yang J. (2016). Emerging role for RBM20 and its splicing substrates in cardiac function and heart failure. Curr Pharm Des.

[18] Sedaghat-Hamedani F., Haas J., Zhu F., Geier C., Kayvanpour E., Liss M. (2017). Clinical genetics and outcome of left ventricular non-compaction cardiomyopathy. Eur Heart J.

[19] Weigand J.E., Boeckel J.N., Gellert P., Dimmeler S., Preiss T. (2012). Hypoxia-induced alternative splicing in endothelial cells. PLoS One.

[20] van den Hoogenhof M.M.G., Beqqali A., Amin A.S., van der Made I., Aufiero S., Khan M.A.F. (2018). *RBM20* mutations induce an arrhythmogenic dilated cardiomyopathy related to disturbed calcium handling. Circulation.

[21] Brodehl A., Ferrier R.A., Hamilton S.J., Greenway S.C., Brundler M.A., Yu W. (2016). Mutations in *FLNC* are associated with familial restrictive cardiomyopathy. Hum Mutat.

[22] Nallari P., Tanjore R., RangaRaju A., Vadapalli S., Remersu S., Narsimhan C. (2010). Genetic variations of *β-MYH7* in hypertrophic cardiomyopathy and dilated cardiomyopathy. Indian J Hum Genet.

[23] Gerstberger S., Hafner M., Tuschl T. (2014). A census of human RNA-binding proteins. Nat Rev Genet.

[24] Richardson P., McKenna W., Bristow M., Maisch B., Mautner B., O’Connell J. (1996). Report of the 1995 World Health Organization/International Society and Federation of Cardiology Task Force on the definition and classification of cardiomyopathies. Circulation.

[25] GTEx Consortium; Laboratory, Data Analysis & Coordinating C (LDACC)—Analysis Working Group; Statistical Methods groups—Analysis Working Group; Enhancing GTEx (eGTEx) groups; NIH Common Fund; NIH/NCI; et al. Genetic effects on gene expression across human tissues. Nature 2017;550:204–13. 10.1038/nature24277 PMC5776756 29022597

[26] Dauksaite V., Gotthardt M. (2018). Molecular basis of titin exon exclusion by RBM20 and the novel titin splice regulator PTB4. Nucleic Acids Res.

[27] Ghanbari M., Ohler U. (2020). Deep neural networks for interpreting RNA-binding protein target preferences. Genome Res.

[28] Danilenko M., Dalgliesh C., Pagliarini V., Naro C., Ehrmann I., Feracci M. (2017). Binding site density enables paralog-specific activity of SLM2 and Sam68 proteins in *Neurexin2* AS4 splicing control. Nucleic Acids Res.

[29] Merkin J., Russell C., Chen P., Burge C.B. (2012). Evolutionary dynamics of gene and isoform regulation in mammalian tissues. Science.

[30] Ding J.H., Xu X., Yang D., Chu P.H., Dalton N.D., Ye Z. (2004). Dilated cardiomyopathy caused by tissue-specific ablation of SC35 in the heart. EMBO J.

[31] Xu X., Yang D., Ding J.H., Wang W., Chu P.H., Dalton N.D. (2005). ASF/SF2-regulated CaMKIIδ alternative splicing temporally reprograms excitation-contraction coupling in cardiac muscle. Cell.

[32] Feng Y., Valley M.T., Lazar J., Yang A.L., Bronson R.T., Firestein S. (2009). SRp38 regulates alternative splicing and is required for Ca^2+^ handling in the embryonic heart. Dev Cell.

[33] Poon K.L., Tan K.T., Wei Y.Y., Ng C.P., Colman A., Korzh V. (2012). RNA-binding protein RBM24 is required for sarcomere assembly and heart contractility. Cardiovasc Res.

[34] Mirtschink P., Krishnan J., Grimm F., Sarre A., Hörl M., Kayikci M. (2015). HIF-driven *SF3B1* induces KHK-C to enforce fructolysis and heart disease. Nature.

[35] Wei C., Qiu J., Zhou Y., Xue Y., Hu J., Ouyang K. (2015). Repression of the central splicing regulator RBFox2 is functionally linked to pressure overload-induced heart failure. Cell Rep.

[36] Ehrmann I., Dalgliesh C., Liu Y., Danilenko M., Crosier M., Overman L. (2013). The tissue-specific RNA binding protein T-STAR controls regional splicing patterns of neurexin pre-mRNAs in the brain. PLoS Genet.

[37] Nguyen T.M., Schreiner D., Xiao L., Traunmüller L., Bornmann C., Scheiffele P. (2016). An alternative splicing switch shapes neurexin repertoires in principal neurons versus interneurons in the mouse hippocampus. Elife.

[38] Ehrmann I., Gazzara M.R., Pagliarini V., Dalgliesh C., Kheirollahi-Chadegani M., Xu Y. (2016). A SLM2 feedback pathway controls cortical network activity and mouse behavior. Cell Rep.

[39] Feracci M., Foot J.N., Grellscheid S.N., Danilenko M., Stehle R., Gonchar O. (2016). Structural basis of RNA recognition and dimerization by the STAR proteins T-STAR and Sam68. Nat Commun.

[40] Esko J.D., Lindahl U. (2001). Molecular diversity of heparan sulfate. J Clin Invest.

[41] Zhu C., Chen Z., Guo W. (2017). Pre-mRNA mis-splicing of sarcomeric genes in heart failure. Biochim Biophys Acta Mol Basis Dis.

[42] Noyes A.M., Zhou A., Gao G., Gu L., Day S., Andrew Wasserstrom J. (2017). Abnormal sodium channel mRNA splicing in hypertrophic cardiomyopathy. Int J Cardiol.

[43] Lara-Pezzi E., Gómez-Salinero J., Gatto A., García-Pavía P. (2013). The alternative heart: impact of alternative splicing in heart disease. J Cardiovasc Transl Res.

[44] Jacob A.G., Smith C.W.J. (2017). Intron retention as a component of regulated gene expression programs. Hum Genet.

[45] Oehler D., Haas J. (2016). Hide and seek: protein-coding sequences inside “non-coding” RNAs. Genomics Proteomics Bioinformatics.

[46] Linke W.A., Kulke M., Li H., Fujita-Becker S., Neagoe C., Manstein D.J. (2002). PEVK domain of titin: an entropic spring with actin-binding properties. J Struct Biol.

[47] Boeckel J.N., Derlet A., Glaser S.F., Luczak A., Lucas T., Heumüller A.W. (2016). JMJD8 regulates angiogenic sprouting and cellular metabolism by interacting with pyruvate kinase M2 in endothelial cells. Arterioscler Thromb Vasc Biol.

[48] Boeckel J.N., Guarani V., Koyanagi M., Roexe T., Lengeling A., Schermuly R.T. (2011). Jumonji domain-containing protein 6 (Jmjd6) is required for angiogenic sprouting and regulates splicing of VEGF-receptor 1. Proc Natl Acad Sci U S A.

[49] Nietsch R., Haas J., Lai A., Oehler D., Mester S., Frese K.S. (2016). The role of quality control in targeted next-generation sequencing library preparation. Genomics Proteomics Bioinformatics.

[50] Schafer S., Miao K., Benson C.C., Heinig M., Cook S.A., Hubner N. (2015). Alternative splicing signatures in RNA-seq data: percent spliced in (PSI). Curr Protoc Hum Genet.

[51] Borchert T., Hübscher D., Guessoum C.I., Lam T.D.D., Ghadri J.R., Schellinger I.N. (2017). Catecholamine-dependent β-adrenergic signaling in a pluripotent stem cell model of takotsubo cardiomyopathy. J Am Coll Cardiol.

[52] Westerfield M. (1995).

[53] Liss M., Radke M.H., Eckhard J., Neuenschwander M., Dauksaite V., von Kries J.P. (2018). Drug discovery with an RBM20 dependent titin splice reporter identifies cardenolides as lead structures to improve cardiac filling. PLoS One.

[54] Meder B., Haas J., Sedaghat-Hamedani F., Kayvanpour E., Frese K., Lai A. (2017). Epigenome-wide association study identifies cardiac gene patterning and a novel class of biomarkers for heart failure. Circulation.

