# Supplementary material for: SLM2 Is A Novel Cardiac Splicing Factor Involved in Heart Failure due to Dilated Cardiomyopathy
Source: Genomics Proteomics Bioinformatics. 2021 Jul 15;20(1):129–46. doi: 10.1016/j.gpb.2021.01.006 (PMC9510876; doi:10.1016/j.gpb.2021.01.006)
Supplement: Supplementary Table S1 [file mmc1.docx]

**Table S1 Primers used for qPCR**

| **Ensembl gene ID** | **Primer name** | **Sequences (5’->3’)** | **Target gene** | **Species** |
| --- | --- | --- | --- | --- |
| ENSG00000089157 | RPLP0-f | GGCGACCTGGAAGTCCAACT | *RPLP0* | Human |
|  | RPLP0-r | CCATCAGCACCACAGCCTTC | *RPLP0* | Human |
| ENSG00000131773 | SLM2-f | CTCATTGAAGTGTTTGCCCCA | *SLM2* | Human |
|  | SLM2-r | TGTGCTTGCCTGATCTCATC | *SLM2* | Human |
