# Supplementary material for: SLM2 Is A Novel Cardiac Splicing Factor Involved in Heart Failure due to Dilated Cardiomyopathy
Source: Genomics Proteomics Bioinformatics. 2021 Jul 15;20(1):129–46. doi: 10.1016/j.gpb.2021.01.006 (PMC9510876; doi:10.1016/j.gpb.2021.01.006)
Supplement: Supplementary Table S2 [file mmc2.docx]

**Table S2 Human tissue types and the respective numbers of tissue samples used for calculation of the heart specificity score**

| Tissue type | Sample number |
| --- | --- |
| Adipose - Subcutaneous | N = 420 |
| Adipose - Visceral (Omentum) | N = 235 |
| Adrenal Gland | N = 161 |
| Artery - Tibial | N = 438 |
| Artery - Coronary | N = 142 |
| Artery - Aorta | N = 251 |
| Bladder | N = 13 |
| Brain - Cortex | N = 148 |
| Brain - Cerebellum | N = 163 |
| Brain - Hippocampus | N = 122 |
| Brain - Substantia nigra | N = 86 |
| Brain - Anterior cingulate cortex (BA24) | N = 114 |
| Brain - Frontal Cortex (BA9) | N = 138 |
| Brain - Cerebellar Hemisphere | N = 135 |
| Brain - Caudate (basal ganglia) | N = 157 |
| Brain - Nucleus accumbens (basal ganglia) | N = 144 |
| Brain - Putamen (basal ganglia) | N = 118 |
| Brain - Hypothalamus | N = 121 |
| Brain - Spinal cord (cervical c-1) | N = 87 |
| Brain - Amygdala | N = 99 |
| Breast - Mammary Tissue | N = 222 |
| Cervix - Ectocervix | N = 6 |
| Cervix - Endocervix | N = 5 |
| Colon - Sigmoid | N = 175 |
| Colon - Transverse | N = 212 |
| Esophagus - Muscularis | N = 299 |
| Esophagus - Mucosa | N = 340 |
| Esophagus - Gastroesophageal Junction | N = 177 |
| Fallopian Tube | N = 7 |
| Heart - Atrial Appendage | N = 219 |
| Heart - Left Ventricle | N = 336 |
| Kidney - Cortex | N = 38 |
| Liver | N = 143 |
| Lung | N = 497 |
| Muscle - Skeletal | N = 566 |
| Nerve - Tibial | N = 390 |
| Ovary | N = 112 |
| Pancreas | N = 204 |
| Pituitary | N = 128 |
| Prostate | N = 123 |
| Minor Salivary Gland | N = 71 |
| Skin - Not Sun Exposed (Suprapubic) | N = 273 |
| Skin - Sun Exposed (Lower leg) | N = 468 |
| Cells - Transformed fibroblasts | N = 307 |
| Small Intestine - Terminal Ileum | N = 106 |
| Spleen | N = 121 |
| Stomach | N = 211 |
| Testis | N = 209 |
| Thyroid | N = 437 |
| Uterus | N = 93 |
| Vagina | N = 99 |
| Whole Blood | N = 1822 |

*Note*: Data were extracted from the GTEx database (https://gtexportal.org/home/).
